# Supramolecular Nested Microbeads as Building Blocks for Macroscopic Self‐Healing Scaffolds

**DOI:** 10.1002/anie.201711522

**Published:** 2018-02-21

**Authors:** Ziyi Yu, Ji Liu, Cindy Soo Yun Tan, Oren A. Scherman, Chris Abell

**Affiliations:** ^1^ Department of Chemistry University of Cambridge Lensfield Road Cambridge CB2 1EW UK; ^2^ Melville Laboratory for Polymer Synthesis Department of Chemistry University of Cambridge Lensfield Road Cambridge CB2 1EW UK; ^3^ Faculty of Applied Sciences Universiti Teknologi MARA 94300 Kota Samarahan Sarawak Malaysia

**Keywords:** cucurbit[8]uril, microbeads, microfluidics, self-healing properties, supramolecular chemistry

## Abstract

The ability to construct self‐healing scaffolds that are injectable and capable of forming a designed morphology offers the possibility to engineer sustainable materials. Herein, we introduce supramolecular nested microbeads that can be used as building blocks to construct macroscopic self‐healing scaffolds. The core–shell microbeads remain in an “inert” state owing to the isolation of a pair of complementary polymers in a form that can be stored as an aqueous suspension. An annealing process after injection effectively induces the re‐construction of the microbead units, leading to supramolecular gelation in a preconfigured shape. The resulting macroscopic scaffold is dynamically stable, displaying self‐recovery in a self‐healing electronic conductor. This strategy of using the supramolecular assembled nested microbeads as building blocks represents an alternative to injectable hydrogel systems, and shows promise in the field of structural biomaterials and flexible electronics.

The construction of self‐healing materials has been driven by developments in materials science and biotechnology with a view to environmental sustainability.^1^, ^2^, ^3^, ^4^, ^5^, ^6^, ^7^, ^8^, ^9^, ^10^, ^11^ To develop maintenance‐free self‐healing structures, the ideal materials should be liquid‐like before processing and then be converted into a free‐standing solid‐like network.^12^, ^13^, ^14^, ^15^ In such systems, the liquid‐like phase brings precursors into place by injection or direct moulding. Once solidification is triggered, the structure retains the chosen morphology but has the ability to recover local mechanical damage, leading to an extension of lifetime.

Recently, intensive developments towards self‐healing materials have been devoted to applying either intrinsic self‐healable units or a pre‐embedded healing agent.^16^, ^17^, ^18^, ^19^, ^20^, ^21^, ^22^, ^23^, ^24^ Intrinsic self‐healing behaviour relies on the introduction of reversible non‐covalent interactions. Typically, building blocks are linked to create diverse supramolecular assemblies upon equilibration between each component, imparting self‐healing adaptability.^16^, ^17^, ^18^, ^19^, ^20^, ^21^ The reversible interactions in supramolecular structures enable materials to flow for injectable delivery under applied shear stress, followed by solidification once the stress is relaxed or removed. The pseudoplastic deformation of supramolecular assemblies depends on the association constant between the complementary molecules. A stronger binding constant often offers the advantages of a faster recovery process, but restrains the ability to flow after injection because the supramolecular materials may transform into the solid‐like phase too quickly to fill the predesigned mould.^14^ While applying a pre‐embedded healing agent by encapsulation would increase the ability to flow during the fabrication process, the extrinsic self‐healing behaviour would be limited by the shelf life, healing cycle, and healing efficiency.^22^, ^23^, ^24^

To simultaneously address the requirements for injectable delivery and fast self‐healing, we have developed a strategy based on the use of supramolecular nested microbeads for the fabrication of mouldable self‐healing materials. Our approach is predicated on the isolation of a pair of complementary polymers in core–shell‐structured microbeads, where the shell temporarily shields a polymer payload from the exterior complementary polymer. This core–shell structure is tailored to deactivate the intermolecular complexation between the two polymers. In comparison with a bulk supramolecular complex, the particulate morphology and low viscosity of microbead dispersions promote the flow for injection.^25^, ^26^, ^27^ Upon release of the polymer payloads by thermal treatment, the paired complementary polymers come into contact as host–guest partners, resulting in dynamic gelation into a moulded shape (Figure 1 a). Solidification is triggered by a stimulus that is easy to control, so that we envisage that it will offer an alternative way to construct supramolecular assemblies with designed morphologies.

**Figure 1:**
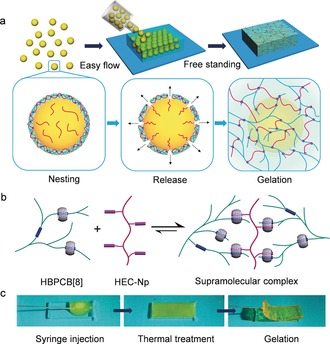
a) Formation of mouldable self‐healing scaffolds from supramolecular assembled microbeads. b) Supramolecular assembly between cucurbit[8]uril‐threaded highly branched polyrotaxanes (HBPCB[8]) and naphthyl‐functionalised hydroxyethyl cellulose (HEC‐Np). c) Photographs of the mouldable transition from a microbead suspension to a macroscale scaffold upon thermal treatment.

In this work, the microbeads were formed from a new class of supramolecular polymer networks assembled from cucurbit[8]uril‐threaded highly branched polyrotaxanes (HBPCB[8]) and naphthyl‐functionalized hydroxyethyl cellulose (HEC‐Np), based on CB[8]‐mediated molecular recognition. CB[8] is a macrocyclic host molecule that is capable of simultaneously encapsulating two guests within its cavity, forming a robust heteroternary host–guest complex.^28^, ^29^, ^30^, ^31^, ^32^, ^33^, ^34^ As illustrated in Figure 1 b, the threading of CB[8] host molecules into highly branched polyrotaxane main chains with a first guest of viologen (MV) provides multiple host units in each macromolecular chain of HBPCB[8]. By mixing with HEC‐Np, the two polymers are joined into a supramolecular copolymer owing to the formation of the CB[8]/viologen/naphthyl heteroternary complexes.^35^ The interaction for the linkage of HBPCB[8] and HEC‐Np is reversible, thus the gel‐like scaffold formed from the microbeads is dynamically stable and self‐healable (Figure 1 c).

To prepare the core–shell‐structured supramolecular host–guest microbeads, a microfluidic device was used to generate uniform water‐in‐oil microdroplets containing supramolecular building blocks. As shown in Figure 2 a, the two aqueous solutions were introduced from their respective inlets [inlets 1 for the mixture of HBPCB[8] and HEC‐Np (1.3 MDa); inlet 2 for HEC‐Np (90 kDa)] and came into contact with each co‐streaming over the nozzle channel. The initial concentrations of HBPCB[8], HEC‐Np (1.3 MDa), and HEC‐Np (90 kDa) in each flow were 1.6 mg mL^−1^, 2.3 mg mL^−1^, and 36.4 mg mL^−1^, respectively. At the flow‐focusing junction, the two solutions were segmented into microdroplets by a perpendicular flow of 3M^TM^ Novec^TM^ 7500 perfluorinated oil containing a 3 wt % fluorous surfactant (XL‐01‐171). The carboxylate‐terminated poly(hexafluoropropylene oxide) dopant DuPont^TM^ Krytox 157FS‐L was also added in the perfluorinated oil phase, giving rise to a negatively charged interface. The HBPCB[8] was labelled with fluorescein (*λ*_ex_=488 nm, *λ*_em_=500–535 nm) for tracking the distribution in microdroplets, while HEC‐Np was labelled with rhodamine B (*λ*_ex_=543 nm, *λ*_em_=565–595 nm).

**Figure 2:**
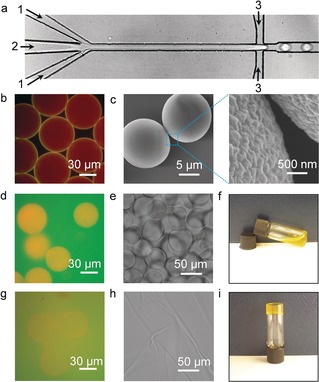
a) Optical micrograph of the generation of microdroplets in a microfluidic device with a flow‐focusing geometry. Inlets 1: mixture of HBPCB[8] and HEC‐Np (1.3 MDa); inlet 2: HEC‐Np (90 kDa), inlet 3: 3M^TM^ Novec^TM^ 7500 perfluorinated oil. b) Fluorescence micrograph of monodisperse microdroplets, illustrating the formation of core–shell‐structured microbeads. c) Scanning electron microscopy (SEM) images of the microbeads upon freeze‐drying. d) Fluorescence image and e) micrograph image of microbeads dispersed in an aqueous phase. f) Photograph of the fluid‐like aqueous microbead dispersion. g) Fluorescence image and h) micrograph image of the annealed microbead sample. i) Photograph of the annealed solid‐like microbeads.

A fluorescence image of the microdroplets showed a core–shell‐structured microbead made from the supramolecular self‐assembled network (Figure 2 b). Yellow fluorescence from the merged red and green fluorescence was clearly resolved on the outer layer of the microdroplets, illustrating the formation of a supramolecular hydrogel shell consisting of HBPCB[8] and HEC‐Np. The accumulation of the supramolecular complex at the interface was attributed to the electrostatic interaction between the negatively charged Krytox surfactant in oil and HBPCB[8] in water, where HBPCB[8] is selectively partitioned to the microdroplet interface for subsequent supramolecular cross‐linking. The red fluorescence dispersed throughout the interior of the microdroplets (Figure 2 b) reveals that the excess of rhodamine B‐labelled HEC‐Np (Np/CB[8]=17 mol mol^−1^) was successfully encapsulated in the microdroplets. Compared to the collapsed structure of the supramolecular microcapsule made from a 1:1:1 mixture of CB[8], MV, and Np (Figure S4), the microdroplets containing free‐standing HEC‐Np polymers yielded solid microbeads upon evaporation. This is shown in Figure 2 c, where each microbead shows spherical morphology with a rough surface due to the supramolecular shell coating.

The network of the supramolecular shell is dynamic and allows for the microbead building blocks to be transformed from their free injectable status into solid‐like scaffolds. To illustrate this, microbeads were rehydrated in HBPCB[8] solution and then subjected to an annealing process, where HBPCB[8] was labelled with fluorescein for fluorescence imaging. As shown in Figure 2 d, osmotic pressure resulted in swelling of the microbeads after rehydration while the surrounding solution maintained its green fluorescence, showing that the supramolecular hydrogel shell was still able to retain and protect encapsulated HEC‐Np. As a result, microbeads dispersed in aqueous media flow easily (Figure 2 e). An inverted vial test clearly shows negligible interaction between the microbeads and their surrounding complementary HBPCB[8] polymer (Figure 2 f). Upon heating rehydrated microbead dispersions at 65 °C for 30 min after rehydration, the green fluorescence surrounding the microbeads changed to yellow owing to mixing with the red fluorescent HEC‐Np (Figure 2 g). This shows that thermal treatment disrupts the host–guest hydrogel shell, triggering the release of HEC‐Np. When the temperature was lowered to 25 °C, the strong association between HBPCB[8] and released HEC‐Np led to a dramatic increase in solution viscosity because of the formation of the supramolecular network (Figure 2 h, i).

To further quantify the thermally induced rheological changes of the supramolecular nested microbeads, their rheological behaviour was studied by comparing their storage (*G*′) and loss (*G*′′) moduli as a function of frequency at 1 % strain (Figure 3 a). For microgels dispersed in HBPCB[8] solution before thermal annealing, *G*′ was lower than *G*′′ up to intermediate angular frequencies, a representative feature of liquid‐like response (Figure 3 a). The microstructure observed by SEM (Figure 2 d) clearly shows a distinct two‐phase composite, with the microbeads distributed homogeneously within the continuous HBPCB[8] phase. Upon annealing, a liquid‐to‐solid transition occurred, as shown in Figure 3 a, with *G*′>*G*′′ over the whole frequency range, indicating the formation of a stable and homogeneous macroscopic hydrogel network. This finding was also supported by SEM analysis (Figure 3 e). According to the steady‐shear rheology measurements (viscosity vs. shear stress) depicted in Figure 3 b, the shear‐insensitive dispersed microbead samples displayed Newtonian behaviour with very low zero‐shear viscosity. Upon annealing, the zero‐shear viscosity of the samples increased by more than three orders of magnitude with shear‐thinning properties. The hydrogel scaffold displayed a broad linear viscoelastic region (Figure S7), before breaking down at strain amplitudes above 100 %.

**Figure 3:**
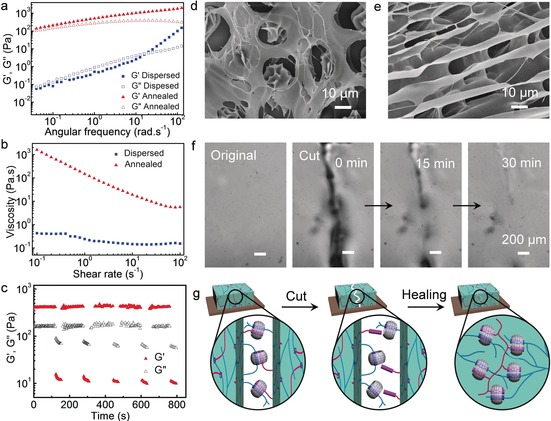
a) Dynamic storage (*G*′) and loss moduli (*G*′′) of samples of dispersed supramolecular microbeads and annealed microbeads (frequency from 0.01 to 200 rad s^−1^, 1 % strain). b) Steady‐shear rheological analysis of the supramolecular microbeads before and after annealing. c) *G*′ and *G*′′ values of the annealed microbeads in a continuous step‐strain measurement (high‐amplitude oscillatory parameters: *γ*=1000 %, *ω*=10 rad s^−1^; low‐amplitude oscillatory parameters: *γ*=0.1 %, *ω*=10 rad s^−1^). SEM images of d) the microbeads in a HEC‐Np matrix and e) the annealed microbeads. f) Micrograph images of the self‐healing process of the hydrogel scaffolds. g) The self‐healing mechanism by dynamic CB[8]‐mediated host–guest chemistry.

The macroscopic self‐healing abilities of the annealed hydrogel scaffolds were investigated by microscopic observations upon a cut. As shown in Figure 3 f, a piece of macroscopic scaffold was cut into two parts with a razor blade, then left for an interval for the self‐healing process to occur in a humid environment at room temperature. An approximately 50 % decrease in the cut size was detected within 15 min, and a fully healed surface formed in 30 min. Microscopic self‐healing/recovery was also detected in a step‐strain rheological test, in which the destruction of the network was induced by a large‐amplitude oscillatory sweep (*γ*=1000 %; Figure 3 c). In this case, *G*′ dramatically decreased from 400 Pa to 12 Pa, resulting in a quasi‐liquid‐state system (tan *δ*=*G*′′/*G*′≈6.7). In the subsequent small‐amplitude oscillatory sweep (*γ*=0.10 %), *G*′ immediately recovered its initial value, and the system returned to a gel state (tan *δ*≈0.4). This process was repeatable for at least five cycles without an appreciable decrease in the average magnitudes of the moduli. Both the macroscopic healing properties and the molecular recovery of the annealed hydrogel scaffold were attributed to the dynamic interactions by CB[8]‐based complementary inclusion complexation (Figure 3 g).

Having shown that supramolecular shielded microbeads can be annealed into a microscopic hydrogel scaffold with self‐healing properties, their utility was demonstrated for self‐healable electronic conductors. This was motivated by the requirement to reduce the failure of electronic devices that is due to mechanical fracture, thereby increasing their lifetime and reliability.^36^ While considerable effort has been invested in covering a conductive nanowire layer (silver nanowires or nanotubes) with self‐healing materials,^37^, ^38^, ^39^, ^40^ the direct application of a supramolecular complex has remained difficult because the viscosity of the assemblies has been too high for infiltration into the nanowire layers. By using liquid‐like suspensions containing supramolecular shielded microbeads, it was possible to obtain a healable conductor with enhanced adhesion between the silver nanowires and the microscopic hydrogel scaffold. As illustrated in Figure 4 a, the process started with drop‐casting a layer of silver nanowires onto a polydimethylsiloxane (PDMS) substrate, followed by coating of the silver nanowires with the microbeads. Upon annealing at 65 °C under vacuum, the microbeads formed a supramolecular composite film in which the silver nanowires were embedded. The conductivity of the resulting composite film depends on the coating of the silver nanowires, and the healable properties should be ensured by the reversible non‐covalent interactions in the CB[8]‐mediated supramolecular matrix.

**Figure 4:**
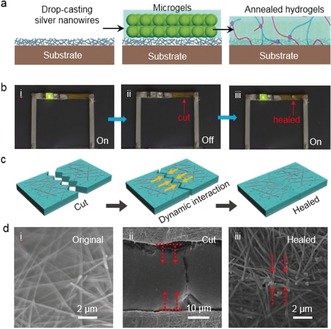
a) Fabrication of a self‐healable electronic conductor using mouldable hydrogel scaffolds. b) Photographs of an LED integrated with silver nanowires/supramolecular conductor for self‐healing tests. c) The healing of silver nanowires by dynamic interactions. d) SEM images of a silver nanowire/supramolecular conductor: i) original one, ii) after being cut, and iii) after healing.

After peeling off the dried silver nanowire/supramolecular composite film from the PDMS substrate, it was connected to a light‐emitting diode (LED) and a power source (Figure 4 b). The LED was powered with a DC voltage of 3 V because of the electrical conductivity of the silver nanowires (Figure 4 b, i). When the silver nanowire/supramolecular composite film was cut, the LED went off. It then re‐lit when a drop of deionized water was applied on the cut (Figure 4 b, ii and b, iii). The restoration of conductivity was attributed to the reconnection of the silver nanowires (Figure 4 c). The SEM images in Figure 4 d were taken to investigate the healing process. Before cutting, a high density of wire–wire junctions between the silver nanowires were bonded by the supramolecular composite, yielding electrical conductivity (Figure 4 d, i). When a cut was made across the surface layer, the silver nanowires were broken (Figure 4 d, ii). Adding a drop of water rehydrated the supramolecular network, resulting in the re‐contact of the silver nanowires through host–guest assembly and self‐healing of the cut (Figure 4 d, iii).

In summary, we have demonstrated a strategy for the development of mouldable self‐healing materials by combining a flowable solution of microbeads and supramolecular chemistry. A droplet‐based microfluidic approach was used for the generation of core–shell‐structured microbeads that could isolate a pair of complementary polymers. The high flow rate of the microbeads enabled their injection into a predesigned mould. Subsequent release of the complementary polymers upon heating triggered supramolecular gelation. The resulting materials were free‐standing and exhibited autonomic healing properties on account of the CB[8]‐based host–guest dynamic interaction. Moreover, the injectable microbeads were attached to silver nanowires to make self‐healable electronic conductors. This is a general method for the generation of materials capable of injectable delivery and fast self‐healing. We anticipate that other supramolecular polymer systems will also benefit from this strategy.

## Conflict of interest

The authors declare no conflict of interest.

## Supporting information

As a service to our authors and readers, this journal provides supporting information supplied by the authors. Such materials are peer reviewed and may be re‐organized for online delivery, but are not copy‐edited or typeset. Technical support issues arising from supporting information (other than missing files) should be addressed to the authors.

Supplementary
