# Supplementary material for: Supramolecular Nested Microbeads as Building Blocks for Macroscopic Self‐Healing Scaffolds
Source: Angew Chem Int Ed Engl. 2018 Feb 21;57(12):3079–83. doi: 10.1002/anie.201711522 (PMC5915745; doi:10.1002/anie.201711522)
Supplement: Supplementary file 1 — Supplementary [file ANIE-57-3079-s001.pdf]

Angewandte Chemie
Eine Zeitschrift der Gesellschaft Deutscher Chemiker

Supporting Information

Supramolecular Nested Microbeads as Building Blocks for Macroscopic Self-Healing Scaffolds

Ziyi Yu, Ji Liu, Cindy Soo Yun Tan, Oren A. Scherman, and Chris Abell**

anie_201711522_sm_miscellaneous_information.pdf

Materials and characterisation

All starting materials were purchased from Sigma-Aldrich and used as received, unless stated otherwise. CB[8] was prepared as documented previously.[1, 2] Fluorous surfactant XL-01-171 was received a kind gift from Dr Xin Li, Sphere Fluidics Ltd. All aqueous solutions were made in deionized water treated with a Milli-Q™ reagent system (resistivity of 18.2 MΩ·cm at 25 °C). Images of droplet formation were obtained using a Phantom v7.2 camera attached to an Olympus IX71 inverted microscope. Microscopic images and fluorescence images were obtained using an Olympus IX81 inverted optical microscope coupled with a camera of Andor Technology EMCCD iXonEM+ DU 897. Scanning electron microscopy (SEM) observation of freeze-dried samples was carried out by using a Leo 1530 variable pressure SEM with an InLens detector. ^{1}H NMR spectra (500 MHz) were collected on a Bruker Avance QNP 500 MHz ultrashield spectrometer, equipped with a 5-mm BBO ATM probe with a z-gradient. Rheological characterisation was performed using a controlled stress Discovery Hybrid Rheometer (DHR-2) from TA Instruments, fitted with a 20-mm parallel plate and the results were analysed using TA Instruments' TRIOS software. The gap in the setup for rheological testing of the samples was set at 0.5 mm and experiments were conducted at 25 ºC. Dynamic oscillatory strain amplitude sweep measurement was conducted at an angular frequency of 10 rad/s. Dynamic oscillatory frequency sweep measurement was conducted at 1% strain amplitude, between 0.01 to 100 rad/s. Step-strain measurement was performed at room temperature to investigate the recovery properties of macroscopic scaffolds upon destruction at high strains (γ = 1000%), followed by a low magnitude strain (γ = 0.1%) to monitor the recovery.

Synthesis

Synthesis of cucurbit[8]uril-threaded highly-branched polyrotaxanes (HBPCB[8])

Firstly, a bifunctional styrenic monomer containing a viologen derivative, StMVSt, was synthesized in the following steps: under nitrogen atmosphere, 4-chloromethyl styrene (6 eq) and 4,4'-bipyridine (1 eq) were dissolved in anhydrous dimethylformamide (DMF) in a round-bottom ask. After heating at 60 ºC for 24 h, the reaction mixture was filtered out. The filtrate was washed with anhydrous DCM, yielding a yellow solid after drying under vacuum at R.T (82%). ^{1}H NMR spectra (D_2O, 500 MHz, 298 K, δ, ppm) = 9.07 (d, 4H), 8.44 (d, 4H), 7.52 (d, 4H), 7.42 (d, 4H), 6.73 (m, 2H), 5.83 (m, 4H), 5.80 (s, 2H) and 5.30 (d, 2H).

Next, a highly branched HBPCB[8] was synthesized through a semi-batch reversible addition−fragmentation chain-transfer (RAFT) polymerisation strategy. A mixture of StMVSt and CB[8] (1:1 mol) aqueous solution was continuously fed into the reaction system at a constant rate during the polymerisation. At the beginning, *N*-hydroxylethyl acrylamide (HEAm) and chain transfer agent 3-benzyltrithiocarbonyl propionic acid (BCPA) were added into a flask in a molar ratio of 50:1. After N_2 was bubbled into the solution for 30 min, the system was heated to 70 ºC. A certain amount of StMVSt and CB[8] (1:1 mol) mixture was fed to the system at a constant rate. At the end of the StMVSt and CB[8] addition, the polymerisation was continued at 70 ºC for another 2 hours, prior to quenching with liquid nitrogen. The polymer product was precipitated into excessive amount of acetone, and then dialysed in a Spectra/Pro dialysis membrane (MWCO 6,000 Da) against 1-adamantane amine aqueous solution for 2 days, and subsequently milli-Q water for 3

days. Purified polymer was then freeze-dried, yielding a yellow amorphous solid (90%). 1H NMR spectra (D_2O, 500 MHz, 298 K, δ, ppm) = 9.70 - 8.50 (m), 7.65 - 6.80 (m), 6.00 -5.25 (m, CB[8]), 4.50 - 3.95 (m, CB[8]), 4.00 - 3.00 (m), 2.50 - 1.00 (m).

For fluorescein-labeled HBPCB[8], HBPCB[8] polymer (0.4 g) and fluorescein isocyanate (0.1 mol.% of hydroxyl group in HBPCB[8]) was dissolved in DMSO, and reacted for overnight with dibutyltin dilaurate (TDL) as catalysis. The crude product was purified through 3 days dialysis (molecular weight cutoff: 6,000 Da), followed by freeze-drying (95%).

Synthesis of naphthyl-functionalised hydroxyethyl cellulose (HEC-Np)

To a solution of HEC (1.3 MDa, 1g) in 120 mL *N*-methylpyrrolidone, 2-naphthyl isocyanate (29.7 mg, 0.18 mmol) and dibutyltin dilaurate (3 drops) was added, and the mixture was stirred for 24 h at room temperature. The product was obtained by precipitation into excessive amount of acetone for three times, and then dried overnight under vacuum at 60 ºC (1.01 g, 98%). ^{1}H NMR spectra (D_2O, 500 MHz, 298 K, δ, ppm) = 7.99 - 7.29 (br, Np-H), 4.50 - 2.90 (br, cellulose backbone).

For the synthesis of rhodamine B-labeled HEC-Np, rhodamine B isocyanate (1 mg, 2 μmol) was added during the reaction between the reaction of HEC and 2-naphthyl isocyanate, following the above-mentioned protocol.

Experimental methods

Microfluidic device fabrication

The microfluidic device for producing water-*in*-oil microdroplets was produced *via* soft lithography by pouring poly(dimethylsiloxane) (PDMS) along with crosslinker (Sylgard 184 elastomer kit, Dow Corning, pre-polymer: crosslinker = 10 : 1) onto a silicon wafer patterned with SU-8 photoresist.[3-4] The PDMS was allowed to solidify at 70 °C overnight before it was peeled off, while inlets and outlets were generated using a biopsy punch. The enclosed microfluidic channels were formed by attaching the moulded PDMS replica onto microscope slides after exposure to oxygen plasma for 8 s in a Femto plasma cleaner. To render the devices hydrophobic and suitable for water-*in*-oil emulsification, 200:1 Novec HFE-7500 (3M) Engineered Fluid oil and trichloro(1H,1H,2H,2H-perfluorooctyl)silane was injected into the microchannels of sealed devices, allowed them to coat on the surface of microchannels at 70 °C overnight. The dimension of the flow-focusing junction in the microfluidic device is 40x40 μm with the channel height of 50 μm.

Droplets-based microfluidic formation of supramolecular shielded microgels

The formation of supramolecular shielded microgels was achieved by assembly of HBPCB[8] and HEC-Np in microfluidic droplets. To generate water-*in*-oil microdroplets, three different liquids were injected into a microfluidic device by three syringe pumps (PHD, Harvard Apparatus) with controlled flow rates (**Figure S1**). One discontinuous aqueous phase for inlet 1 was prepared by dissolving

HBPCB[8] and HEC-Np (1.3 MDa) in water, and another discontinuous phase for inlet 2 was prepared by dissolving HEC-Np (90 kDa) in water. 3M™ Novec™ 7500 perfluorinated oil containing a 3 wt% fluorous surfactant (XL-01-171, kind gift from Dr Xin Li, Sphere Fluidics Ltd) and 1.8 wt% DuPont™ Krytox 157FS-L was used as the continuous phase for inlet 3. The continuous phase and both discontinuous phase solutions were separately loaded into 1 mL syringes before connecting to the microfluidic chip. Syringes with needles were mounted on syringe pumps and fitted with polyethylene tubing, while the other end of the tubing was inserted into the appropriate inlets of a microfluidic chip. Microdroplets formation was initiated by pumping 3M™ Novec™ 7500 perfluorinated oil into the device at the rate of 500 μL/h, following by pumping of mixture of HBPCB[8] and HEC-Np (1.3 MDa) at a speed of 100 μL/h, and HEC-Np (90 kDa) at speed of 150 μL/h. In a typical experiment, the molar concentration of CB[8] was 500 μM, which was equal to the molar concentration of Np in aqueous phase for inlet 1. The molar concentration of Np in aqueous phase for inlet 2 was 8 mM. Upon generation, the microdroplets were either collected in the PDMS reservoir downstream or transferred to a plastic petri dish. Upon collection, water-*in*-oil microdroplets in the PDMS reservoir were placed in the IX81 inverted optical microscope for characterization, while microdroplets in the petri dish were left in a fumehood for evaporation of both perfluorinated oil and water. The final products were washed by 3M™ Novec™ 7100 perfluorinated oil to remove surfactant XL-01-171 and DuPont™ Krytox 157FS-L.

Generation of a macroscopic scaffold from supramolecular shielded microgels

The macroscopic scaffold was prepared by injection of supramolecular shielded microgels suspensions in a pre-designed mould. Supramolecular shielded microgels (19.5 mg) were mixed with HBPCB[8] solution (CB[8] = 8 mM, 500 μL) and loaded in a 1 mL syringe. The mixture was extruded through an 25G needle for injected into a pre-designed mould and filled in the whole cubic area. Macroscopic scaffold annealing was conducted at 65 ^{o}C for 30 min and then cooled down to the room temperature. To prevent evaporation of water during the thermo-treatment, the mould was kept in a petri dish sealed by the parafilm.

Fabrication of self-healable electronic conductor using mouldable hydrogel scaffolds

A silver nanowires dispersion was drop-cast on the precleaned PDMS substrate to form a network of silver nanowires. After drying, a mixture of supramolecular shielded microgels (19.5 mg) in HBPCB[8] solution (CB[8] = 8 mM, 500 μL) was injected on the top of the silver nanowires. The PDMS substrate was subsequently placed in an oven with 65 ^{o}C for 30 min for 6 h. The resulting composite film was peeled off from the PDMS substrate and used as a conductive wire for connecting a light-emitting diode (LED) and a power source.

Supplementary figures

Figure S1 shows the molecular structures of the cucurbit[8]uril-threaded highly-branched polyrotaxanes (HBPCB[8]) and naphthyl-functionalised hydroxyethyl cellulose (HEC-Np), which were used for constructing of supramolecular shielded microgels in microfluidic droplets.

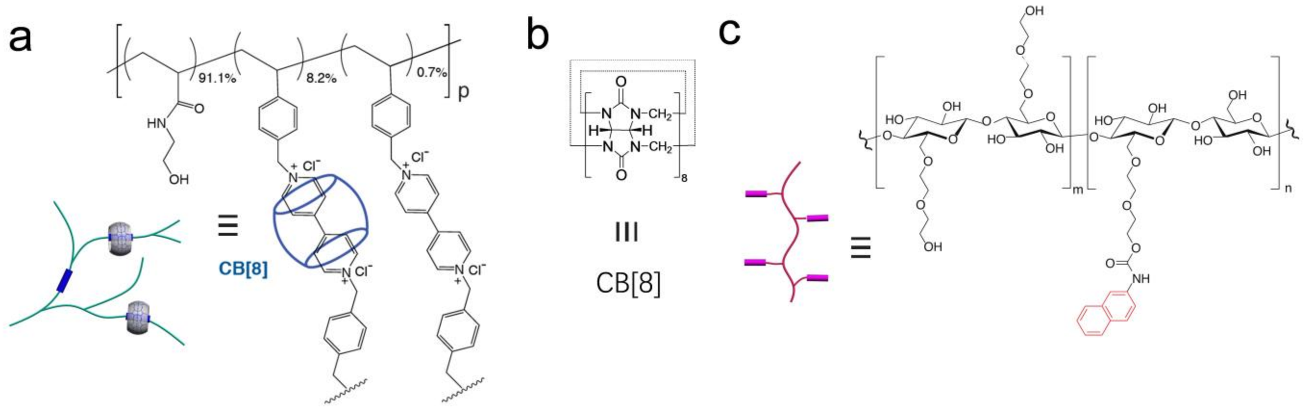

Figure S1. Molecular structure of HBPCB[8], CB[8], and HEC-Np.

1H NMR Spectroscopy (D_2O, 500 MHz, 298 K) (ppm) = 9.70 - 8.50 (m, 9H), 7.65 - 6.80 (m, 21H), 6.00 - 5.25 (m, CB[8]), 4.50 - 3.95 (m, CB[8]), 4.00- 3.00 (m, 75H), 2.50 - 1.00 (m, 110H, polymer backbone), as shown in Figure S2. Zimm plot (H_2O): Mw = 3.0 ± 1.5 MDa, dn/dc = 0.181 mL g^{-1}.

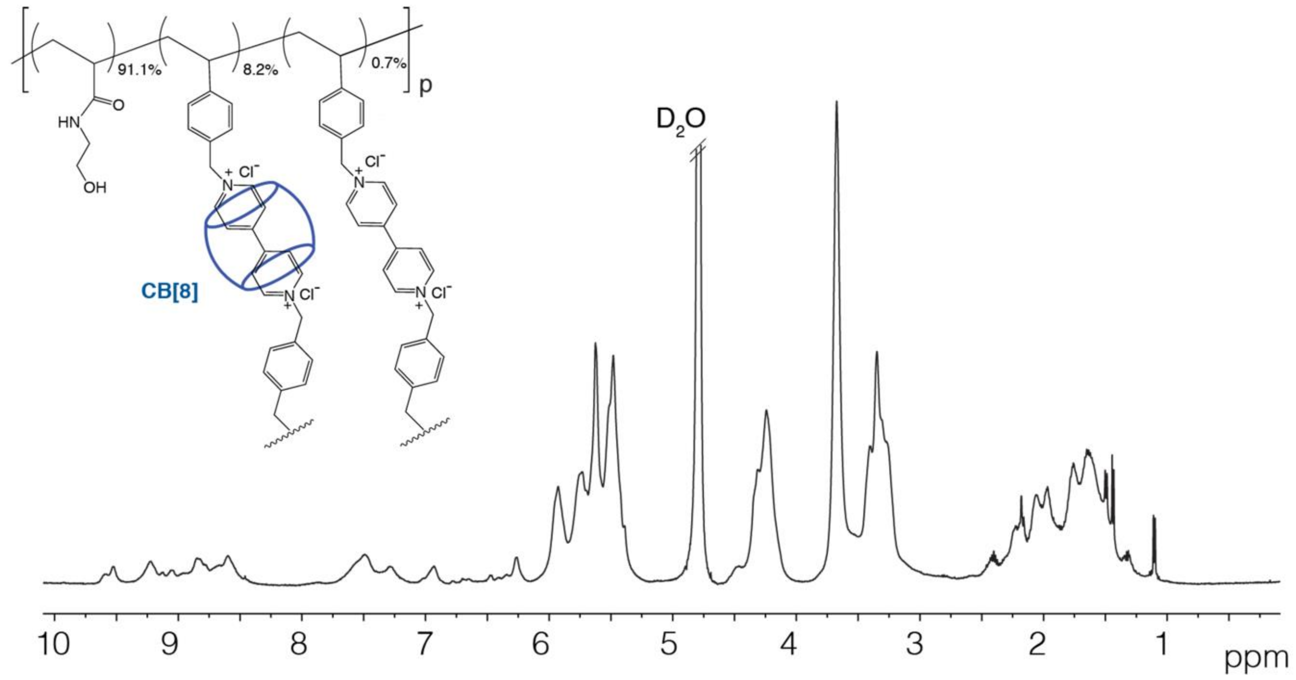

Figure S2. 1H NMR spectrum of the highly branched HBPCB[8] (D_2O, 298 K, 500 MHz).[5]

Microdroplets containing a mixture of HBPCB[8], CB[8], and HEC-Np were genereated by a flow-focusing microfluidic device (Figure S3).

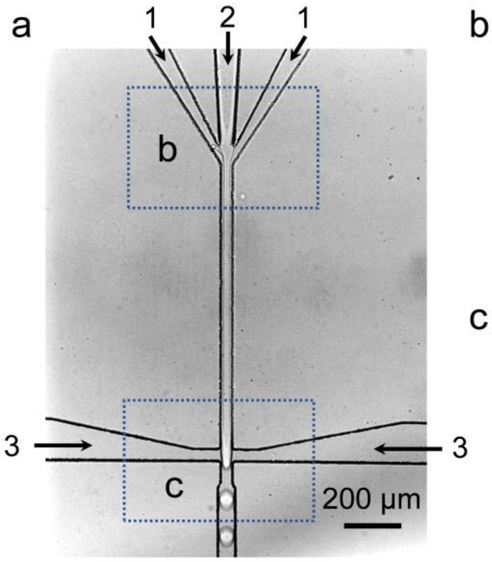

Figure S3. (a) Optical micrograph of the generation of microdroplets in a microfluidic device with a flow-focusing geometry [Inlets 1: mixture of HBPCB[8] and HEC-Np (1.3 MDa); Inlet 2: HEC-Np (90 kDa); Inlet 3: Fluorinert FC-40 perfluorinated oil]. (b, c) Enlarged optical micrograph of the microfluidic device.

Upon water evaporation in microdroplets, it was found that the structure was collapsed because of a lack of internal support, with creases and folds clearly observed on the supramolecular polymer skins (Figure S4).

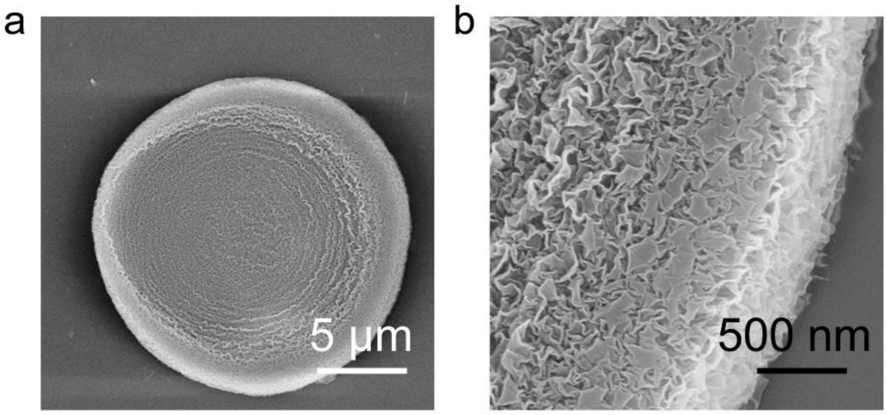

Figure S4. Scanning electron microscopic (SEM) images of the water dehydrated microdroplets made from HBPCB[8] and HEC-Np (1.3 MDa) with a 1:1:1 ratio of CB[8], MV and Np.

Compared to the microdroplets containing only rhodamine B-labeled HEC-Np (90 kDa) (Figure S5a), presence of HBPCB[8] and HEC-Np (1.3 MDa) formed supramolecular skins, and were able to retain the cargo during rehydration (Figure S5b).

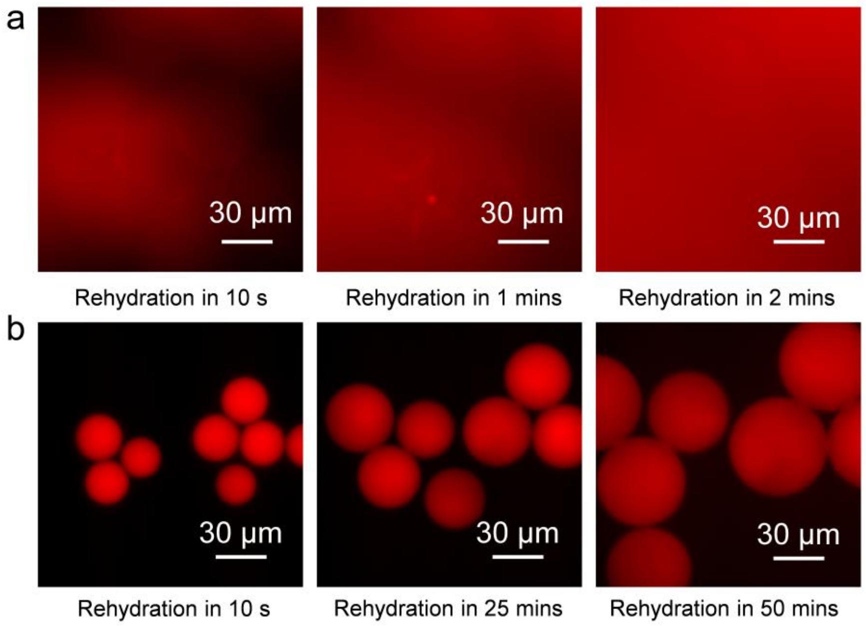

Figure S5. (a) Fluorescent micrographs of the rehydrated microspheres made from rhodamine B-labeled HEC-Np (90 kDa), showing the immediate dissolution of the microspheres upon rehydration. (b) Fluorescent micrographs of the rehydrated supramolecular shielded microgels, showing the supramolecular skin retained the encapsulated rhodamine B-labeled HEC-Np.

The supramolecular shielded microgels maintained the spherical shape after rehydration at 25 ^oC in HBPCB[8] aqueous solution for 50 mins, without any HEC-Np (90 kDa) released into the solution (Figure S6a). On the contrary, thermal treatment of the rehydrated shielded microgels destructed the supramolecular hydrogel skins, activating the releasing of HEC-Np (90 kDa).

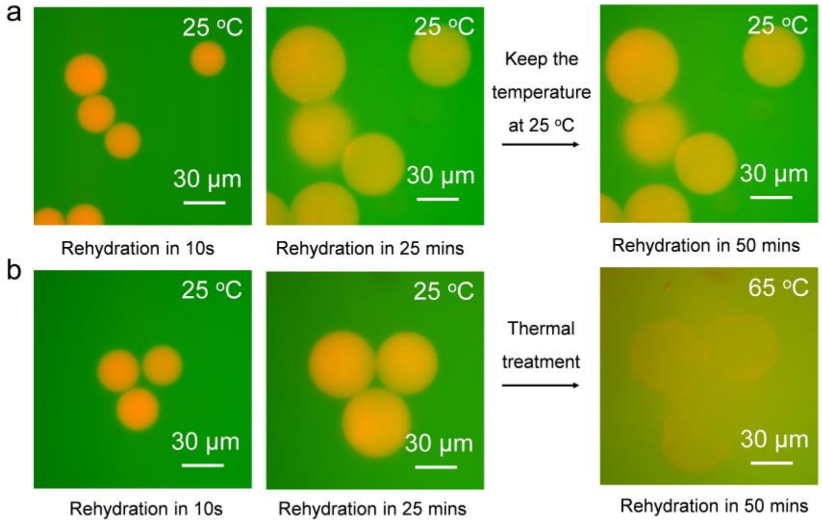

Figure S6. Fluorescent micrographs of the rehydrated supramolecular shielded microgels. (a) Rehydration at 25 ^oC in HBPCB[8] aqueous solution. (b) After rehydration at 25 ^oC in HBPCB[8] aqueous solution for 25 minutes, the temperature was increased at 65 ^oC for incubation another 25 minutes.

In the dynamic rheological measurements, the dynamic amplitude sweep was carried out. A sharp increase in *G*' was observed from *ca*. 2 Pa (dispersed supramolecular microgels) to *ca*. 1000 (annealed microgels samples) upon 30-min annealing at 65 ºC.

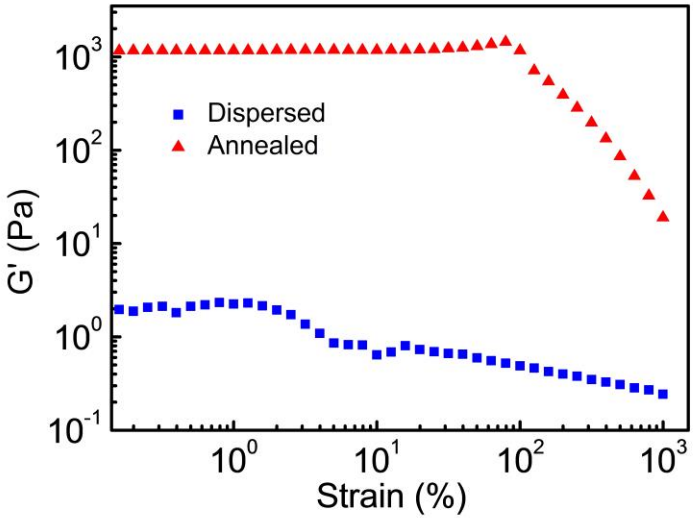

Figure S7. Dynamic amplitude sweeping of the dispersed supramolecular microgels and the annealed microgels samples at frequency of 10 rad/s.

Additional data related to this publication is available at the University of Cambridge data repository (https://doi.org/10.17863/CAM.18195)

References

1. J. Kim, I.-S. Jung, S.-Y. Kim, E. Lee, J.-K. Kang, S. Sakamoto, K. Yamaguchi, K. Kim, *J. Am. Chem. Soc.*, **2000**, 122, 540-541.

2. A. Day, A. P. Arnold, R. J. Blanch, B. Snushall, *J. Org. Chem.*, **2001**, 66, 8094-8100.

3. D. Qin, Y. Xia, G. M. Whitesides, *Adv. Mater.*, **1996**, 8, 917-919.

4. D. C. Duffy, J. C. McDonald, O. J. A. Schueller, G. M. Whitesides, *Anal. Chem.*, **1998**, 70, 4974-4984

5. C. Tan, J. Liu, A. Groombridge, S. Barrow, C. Dreiss, O. A. Scherman, *Adv. Funct. Mater.,* **2017**, DOI: 10.1002/adfm.201702994
